# Micronutrient Deficiencies among Breastfeeding Infants in Tanzania

**DOI:** 10.3390/nu9111258

**Published:** 2017-11-17

**Authors:** Alexandra L. Bellows, Emily R. Smith, Alfa Muhihi, Christina Briegleb, Ramadhani A. Noor, Salum Mshamu, Christopher Sudfeld, Honorati Masanja, Wafaie W. Fawzi

**Affiliations:** 1 Department of Epidemiology, Harvard T.H. Chan School of Public Health, Boston, MA 02115, USA; mina@hsph.harvard.edu; 2 Department of Global Health and Population, Harvard T.H. Chan School of Public Health, Boston, MA 02115, USA; ersmith@hsph.harvard.edu (E.R.S.); chbriegleb@gmail.com (C.B.); crsudfeld@gmail.com (C.S.); 3 Department of Gastroenterology, Hepatology and Nutrition, Boston Children’s Hospital, Boston, MA 02115, USA; 4 Management and Development for Health, P.O. Box 79810 Dar es Salaam, Tanzania; selukundo@gmail.com; 5 Department of Nutrition, Harvard T.H. Chan School of Public Health, Boston, MA 02115, USA; ramanoor@gmail.com; 6 Africa Academy for Public Health, P.O. Box 79810 Dar es Salaam, Tanzania; salum.mshamu@gmail.com; 7 Ifakara Health Institute, P.O. Box 78373 Dar es Salaam, Tanzania; hmasanja@ihi.or.tz

**Keywords:** micronutrients, infants, Tanzania, vitamin B12, vitamin D

## Abstract

Infant mortality accounts for the majority of child deaths in Tanzania, and malnutrition is an important underlying cause. The objectives of this cross-sectional study were to describe the micronutrient status of infants in Tanzania and assess predictors of infant micronutrient deficiency. We analyzed serum vitamin D, vitamin B12, folate, and ferritin levels from 446 infants at two weeks of age, 408 infants at three months of age, and 427 mothers three months post-partum. We used log-Poisson regression to estimate relative risk of being deficient in vitamin D and vitamin B12 for infants in each age group. The prevalence of vitamin D and vitamin B12 deficiency decreased from 60% and 30% at two weeks to 9% and 13% at three months respectively. Yet, the prevalence of insufficiency at three months was 49% for vitamin D and 17% for vitamin B12. Predictors of infant vitamin D deficiency were low birthweight, urban residence, maternal education, and maternal vitamin D status. Maternal vitamin B12 status was the main predictor for infant vitamin B12 deficiency. The majority of infants had sufficient levels of folate or ferritin. Further research is necessary to examine the potential benefits of improving infants’ nutritional status through vitamin D and B12 supplements.

## 1. Introduction

In 2015, the global under-five mortality rate was estimated to be 41 deaths per 1000 livebirths and the neonatal mortality rate was estimated to be 18 deaths per 1000 livebirths; however, many low- and low-middle income countries have significantly higher rates [1]. In order to meet the new Sustainable Development Goals (SDGs), significant reductions in under-five mortality and neonatal mortality must be achieved [2]. Despite progress in reducing child mortality in Tanzania, neonatal and infant mortality still accounts for the majority of child deaths [3,4]. Tanzania’s under-five mortality was 60 deaths per 1000 livebirths and neonatal mortality rate was 22 deaths per 1000 livebirths in 2015 [1]. Understanding the relative importance of underlying causes of neonatal and post neonatal death is important for identifying high impact interventions. 

Malnutrition is a major underlying cause for a significant proportion of under-five mortality [5]. Micronutrient deficiencies are preventable sources of malnutrition that can directly affect an individual’s health and their cognitive and physical attainments. Micronutrients consist of both vitamins and minerals that are vital to biochemical pathways in the body that promote physical growth and cognitive development [6]. An estimated two billion individuals are affected by chronic deficiencies in essential micronutrients [7,8]. Evidence suggests that deficiency of multiple micronutrients tend to coexist in low- and middle-income countries where diets with low dietary diversity are common [9,10,11]; women and children under five years of age are at the highest risk of deficiency [6]. The first 1000 days of life is a critical period for growth and development, yet little is known about the prevalence of micronutrient deficiencies in infants, particularly in infants less than six months of age. 

Vitamin D, vitamin B12, folate, and ferritin are essential for child growth and development. Vitamin D regulates calcium metabolism, promotes bone health, and its deficiency is associated with microbial infections, inflammation, and HIV infection [12]. In addition, it is one of the most common nutritional deficiencies in both children and adults [13]. Vitamin D is naturally occurring in the human body and is synthesized by the body when the skin is exposed to ultraviolet B light. Consumption of food rich in vitamin D has a limited effect on serum vitamin D status [14,15]. Studies have shown that low infant vitamin D status is associated with low birthweight and lack of sunlight [16]. Evidence suggests newborn vitamin D status is highly correlated with maternal vitamin D status [17]. Vitamin B12 deficiency is primarily due to insufficient dietary intake of animal food sources or poor absorption. Vitamin B12 is only naturally found in animal food products and is not synthesized in the body [18]. Populations that consume mostly a vegetarian diet or have infrequent access to animal sources of food are at a high risk of vitamin B12 deficiency [18,19]. Infants aged four-to-six months with low vitamin B12 have a greater risk of developmental delays [20]. Studies have found that women deficient in vitamin B12 have lower concentrations of vitamin B12 in their breastmilk, potentially affecting the vitamin B12 status of their breastfed infant [18,21,22,23]. Studies have shown that premature and low-birth-weight infants have lower levels of vitamin B12 compared to full-term infants and those with normal birth weight [24]. Low folate during infancy is associated with delayed growth and low folate during pregnancy can result in neural tube defects [25]. Folate is introduced into the body through consumption of foods high in dietary folate such as green leafy vegetables, legumes, and fortified cereals. Populations that consume unfortified wheat or rice are at a higher risk of deficiency [18]. Maternal folate status does not affect breastmilk concentration of folate unless the mother is severely deficient [26]. Iron deficiency is a main source of anemia worldwide [27]. Infants with iron deficiency anemia are at a greater risk for impaired motor, mental, and physical development [28]. Ferritin stores in the body are high at birth and continue to rise for the first two months of life and then rapidly decline until the end of the first year [29]. At birth, duration of gestation, sex, and maternal iron status can affect ferritin concentrations [30].

The objectives of this study were to assess vitamin D, vitamin B12, folate, and ferritin status among Tanzanian infants at two weeks and three months of age and assess the association between risk factors, including maternal micronutrient status, with infant micronutrient deficiency. 

## 2. Materials and Methods

### 2.1. Study Participants

Study participants were randomly selected infants and mothers from the Tanzania NEOVITA trial a randomized double-blind, placebo control trial for vitamin A supplementation in the Dar es Salaam and Morogoro Regions of Tanzania. Details of the study have been described elsewhere [31,32]. Briefly, pregnant women were recruited during household visits, at an antenatal care visit, or at birth; newborns were randomized to a single dose of vitamin A (50,000 IU) or placebo within three days of birth. Participants included in the study intended to stay in the study area for at least six months after birth. Infants enrolled in other clinical trials and infants born with congenital abnormalities were excluded from enrollment. Field staff assessed infant health at 1, 3, 6, and 12 months of age. This study received ethical approval from the Institutional Review Boards of the Harvard T.H. Chan School of Public Health, Ifakara Health Institute, National Institute of Medical Research, and by the WHO Ethical Review Committee [31,32]. 

### 2.2. Blood Sampling

A subset of infants was randomly selected for blood sample collection at ages two weeks and three months. For sampling at three months, infants sampled at two weeks were excluded; therefore, no infant was sampled at both two weeks and three months. Maternal samples were collected at three months postpartum concurrent with infant samples at that age. Blood specimens were from a random sampling of participants across all study areas and seasons [32]. Written informed consent was obtained before extracting 0.5 to 1.0 mL of blood via a heel-prick from the infant or via venipuncture from the mother. 

Blood samples were sent to the study laboratory for serum separation and cryostorage at −80 °C. Blood samples were analyzed at Boston Children’s Hospital (Boston, MA, USA) for vitamin D (25OHD), serum vitamin B12, folate, and ferritin [32]. Vitamin D was measured by an enzyme immunoassay from Immunodiagnostic Systems Inc. (Fountain Hills, AZ, USA). Serum vitamin B12 and folate were measured with an electrochemiluminescence binding assay on the Roche E Modular system (Roche Diagnostics, Indianapolis, IN, USA) [33]. Ferritin was measured by a colorimetric assay using the Roche P Modular system (Roche Diagnostics, Indianapolis, IN, USA). High-sensitivity C-reactive protein (hsCRP) was measured with an immunoturbidimetric assay on the Roche P Modular system (Roche Diagnostics, Indianapolis, IN, USA).

### 2.3. Statistical Analysis

Mean, standard deviation, median, 25th percentile, and 75th percentile for each micronutrient were calculated for infants two weeks, infants three months, and mothers three months postpartum. Definitions of deficiency for each micronutrient were based on established cutoffs. Definitions for maternal and infant micronutrient deficiencies are listed in Table 1. 

We used SAS PROC GENMOD’s log-Poisson regression with robust variances [37] to estimate the relative risk of being deficient for vitamin D and vitamin B12 based on both infant and maternal predictors for infants at two weeks and three months old. Outcomes of interest were infant vitamin D deficiency (<50 nmol/L) and infant vitamin B12 deficiency (<203 pg/mL). Risk factors included in the multivariate model were low birthweight (less than 2500 g), preterm birth (less than 37 weeks of gestation), sex, urban residence, number of antenatal care visits (<4 visits, ≥4 visits), maternal education (none, 1–7 years, >7 years), maternal age (<20 years, 20–25 years, 25–30 years, >30 years), and maternal micronutrient status. Urban residence was defined as those recruited from Dar es Salaam study sites. For the model predicting infant vitamin D deficiency at three months, maternal vitamin D status was defined as either sufficient (≥50 nmol/L), deficient (<50 nmol/L), or missing [34]. For the model predicting infant vitamin B12 deficiency at three months, maternal vitamin B12 status was defined as sufficient (>271 pg/mL), marginally deficient (203–271 pg/mL), deficient (<203 pg/mL), or missing [18]. Univariate analyses were conducted for variables that were considered a priori as potential predictors, and all variables were included in the multivariate model. Preterm and low birthweight were not included in the same model due to collinearity. For all covariates besides preterm, reported relative risks, confidence intervals, and *p* values are from a multivariate model that includes low birthweight, but not preterm. *p* for trend values were reported for maternal age and maternal education categorical variables. Models for infants at two weeks did not have maternal vitamin deficiency because maternal status was not collected in this age group. Statistical analysis was conducted using SAS, Version 9.4 (SAS Institute Inc., Cary, NC, USA).

## 3. Results

We processed 1281 blood samples that measured at least one of the following micronutrients: vitamin D, vitamin B12, folate, ferritin, and hsCRP. A total of 446 infants were sampled at two weeks old, 408 infants sampled were at three months old, and 427 mothers were sampled at three months postpartum. Of the infants sampled at three months and mothers sampled at three months postpartum, 364 were infant-mother pairs. 

Baseline characteristics for infants with samples at two weeks and infants with samples at three months are displayed in Table 2. A total of 13% of infants sampled at two weeks and 17% of infants sampled at three months were born preterm (before 37 weeks of gestation). The majority of infants (97.8%) in our sample received colostrum at birth and 90% of infants were breastfed within one hour of birth. From the sample, 43% of mothers reported exclusive breastfeeding at three months and 46% reported partial or predominate breastfeeding at three months. For both infant groups, only a quarter of mothers reported having four or more antenatal care visits (ANC).

**Figure 1:**
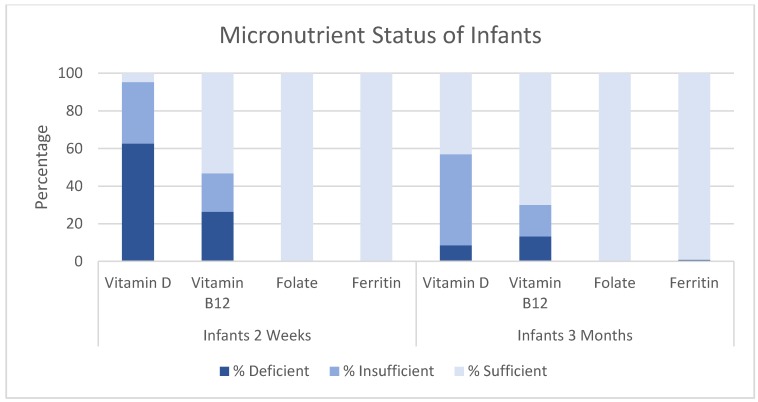
Micronutrient status of infants two weeks and three months of age. Y-axis indicates percentage. X-axis indicates micronutrient.

Descriptive statistics for infants’ and mothers’ micronutrient statuses are displayed in Table 3. On average, infants at three months had higher vitamin D levels (nmol/L), vitamin B12 (pg/mL), folate (ng/mL) levels, and lower ferritin (ng/mL) levels than infants two weeks old. For infants two weeks old, 62.6% were categorized as deficient in vitamin D and 26.4% were deficient in vitamin B12. No infants at two weeks of age had deficiencies in folate or iron. For infants at three months, 8.6% were categorized as deficient in vitamin D and 48.4% were categorized as insufficient deficient in vitamin D. 13.3% of infants at three months were categorized as vitamin B12 deficient and 16.7% were categorized as insufficient in vitamin B12. Less than 1% of infants at three months were categorized as deficient in folate or iron (Figure 1). 

**Figure 2:**
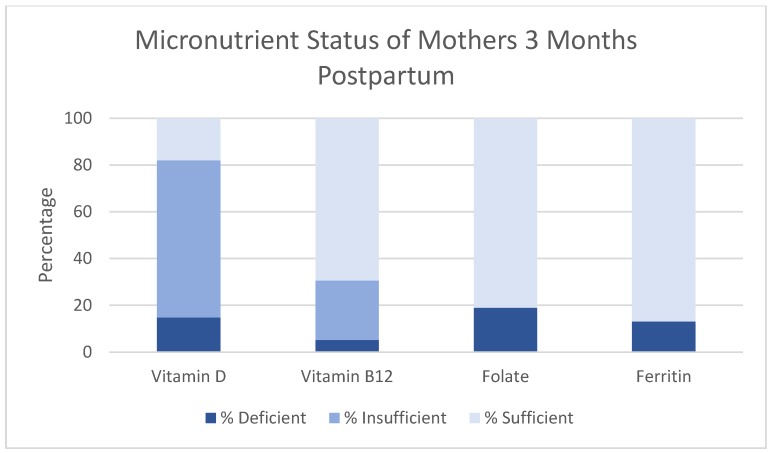
Micronutrient status of mothers three months postpartum. Y-axis indicates percentage. X-axis indicates micronutrient.

For mothers at three months postpartum, 14.8% were deficient in vitamin D and 67.2% were categorized as insufficient; 5.2% of mothers were vitamin B12 deficient, while 25.6% of mothers were categorized as insufficient for vitamin B12; 19.0% of mothers were deficient in folate and 26.0% of mothers were deficient in ferritin (Figure 2).

We identified several predictors of infant vitamin D deficiency at two weeks and three months (Table 4). For infants at two weeks, maternal education level was the main predictor of Vitamin D deficiency. Infants at two weeks whose mothers received at least a secondary education were at twice the risk of being deficient in vitamin D than those whose mothers received no education. For infants at three months, infants who weighed less than 2500 g (low birthweight) at birth had 4.4 times the risk of being deficient in vitamin D compared to infants who weighed more than 2500 g at birth (multivariate Relative Risk (RR): 4.37, 95% Confidence Interval (CI): 1.95, 9.77). Infants at three months living in an urban area had nearly three times the risk of being deficient in vitamin D compared to infants living in rural area (multivariate RR: 2.95, 95% CI: 1.46, 5.97). In addition, infants at three months born to mothers who had some primary education had 5.2 times the risk of being deficient in vitamin D, and infants born to mothers with some secondary education had 8.5 times the risk of being deficient in vitamin D compared to infants whose mothers received no education (*p* for trend: 0.03). Finally, infants at three months whose mothers were vitamin D deficient (<50 nmol/L) had 2.8 times the risk of being deficient in vitamin D compared to infants whose mothers were not deficient in vitamin D (multivariate RR: 2.83, 95% CI: 1.45, 5.52). 

We also found several predictors for infant vitamin B12 deficiency for infants at two weeks and three months of age (Table 5). For infants at two weeks, maternal age was the main predictor of infant vitamin B12 status. As maternal age increased the relative risk of an infant at two weeks being deficient in vitamin B12 increases (*p* for trend = 0.0006), yet for infants at three months increased maternal age potentially decreased risk of deficiency but not a significant predictor of infant vitamin B12 status. For infants at three months, infants whose mothers were marginally vitamin B12 deficient (203–271 pg/mL) had four times the risk of being deficient in vitamin B12 compared to infants whose mothers were not deficient in vitamin B12 (multivariate RR: 4.06, 95% CI:1.88, 8.70). Infants at three months whose mothers were vitamin B12 deficient (<203 pg/mL) had 9.6 times the risk of being deficient in vitamin B12 compared to infants whose mothers were not deficient in vitamin B12 (multivariate RR: 9.63, 95% CI: 4.83, 19.17). 

## 4. Discussion

This study characterized the micronutrient status and identified predictors of micronutrient deficiency for young infants at two weeks and three months. In this Tanzanian cohort of infants and mothers, we found that some degree of maternal micronutrient deficiency was prevalent for all micronutrients assessed in this study. For infants at two weeks and three months, micronutrient deficiency was prevalent only for vitamin D and vitamin B12, but not for folate or iron. Our study found that over 60% of infants at two weeks were deficient in vitamin D. By the age of three months prevalence of vitamin D deficiency drops significantly to approximately 9%, but still greater than 40% of infants at three months were insufficient in vitamin D. Results from the three month cohort are consistent with other studies in Kenya and Uganda that found that a significant proportion of infants and children were at least marginally vitamin D deficient [38,39]. Our study also found that at two weeks of age, nearly 30% of infants were deficient and 20% were insufficient in vitamin B12, while 13% and 17% of infants at three months were vitamin B12 deficient and insufficient, respectively. While there is limited evidence from other studies conducted in East Africa regarding infant vitamin B12 status, these results are consistent with a study in Nepal that found that 17% of infants were vitamin B12 deficient and 40% were marginally deficient [11]. 

Few epidemiological studies have examined micronutrient status of infants less than six months; therefore, little is known about how micronutrient status changes over time during infancy. Although our data is not longitudinal, our results suggest that on average vitamin B12, folate, and vitamin D are lower in the first few weeks of life than at three months of age, while ferritin is higher in the first few weeks of life and then declines. A randomized longitudinal study in New Zealand also found that infant serum vitamin D increases steadily from birth to six months of age in the placebo group [40]. At birth and in early infancy, sources of infant serum vitamin D are primarily from maternal sources of vitamin D and as the child gets older, there is greater opportunity for the infant to synthesize vitamin D from ultraviolet light. The changes in ferritin levels during infancy are well documented in the literature and indicate that ferritin may not be the best measure of iron deficiency during this time period of life unless more precise cutoff points for deficiency are established [41]. Further longitudinal research is necessary to determine normal micronutrient levels in infancy and if low levels of micronutrients at infancy are predictors of deficiency later in life. 

This study reports that infants at three months who were less than 2500 g at birth, residing in an urban area, whose mothers had attained higher levels of education, or whose mothers were vitamin D deficient were more likely to be vitamin D deficient. These results are consistent with other studies that show that infant vitamin D deficiency is associated with low birthweight and maternal vitamin D status [11]. Infants born to mothers with adequate vitamin D levels should have adequate vitamin D levels for their first eight weeks of life [42]. If the mother’s stores are insufficient during pregnancy, infants may be born with low vitamin D levels and vitamin D concentration in breastmilk may be low [40]. The association between infant vitamin D deficiency and higher maternal education and urban residence may be explained by the amount of direct sunlight an infant receives. Unfortunately, data on the amount of direct sunlight was not available for this population, but we hypothesize that infants born to mothers with higher levels of education may be less likely to spend long periods outdoors. Similarly, infants in urban areas may spend less time outdoors than their rural peers. 

Additionally, we found that the main predictor of infant vitamin B12 deficiency was the maternal vitamin B12 status. Infants whose mothers were vitamin B12 deficient had more than nine times the risk of being deficient in vitamin B12, and infants whose mothers were marginally deficient in vitamin B12 had four times the risk of being deficient than infants whose mothers were vitamin B12 sufficient, indicating a dose–response relationship. The only source of vitamin B12 for infants is breastmilk. Vitamin B12 concentrations in breastmilk are dependent on maternal vitamin B12 status [23]. Few studies have examined the effect of vitamin B12 supplementation in pregnancy, but a randomized trial in India found that supplementing women during pregnancy and early lactation significantly increased maternal and infant vitamin B12 serum concentrations [43]. 

Interestingly, while there is significant vitamin D and vitamin B12 deficiency in infants, there are very few cases of folate or iron deficiency in our study cohort. This may be due to the fact that antenatal care recommendations in Tanzania per WHO guidelines provide prenatal supplements with iron and folic acid, but do not provide supplements for vitamin B12 or vitamin D [44]. In 2016, the WHO updated their antenatal care recommendations and now recommend eight antenatal care visits [45]. These new guidelines still recommend routine supplementation for iron folic-acid, but still do not recommend routine supplementation for vitamin D and vitamin B12 during antenatal care visits due to limited evidence currently available. 

This study has a few limitations. First, study participants came from a large randomized trial recruiting from Morogoro and Dar es Salaam study areas, hence the results may not be generalizable to all regions of Tanzania. In addition, micronutrient cutoffs for vitamin D, vitamin B12, and folate are based on cutoff points for adults. These cutoff points may not be most appropriate for young infants. One of the main challenges of our analysis was the lack of well-defined cutoff points for infants at two weeks, and we did not have information about maternal vitamin status at two weeks. Furthermore, only 25% of infants and mothers had hsCRP measured, therefore we could not adjust ferritin levels for acute phase infection. Additionally, we chose to measure serum folate because it is easier to measure in the field, but it is not as robust as red cell folate. In addition, serum folate fluctuates with intake therefore is a better measurement of short-term deficiency, rather than long-term status. Lastly, this was not a longitudinal study. Infants measured at two weeks were not the same infants measured at three months and thus we could not assess changes in micronutrient status over time. Our study has several strengths. Few studies have previously examined multiple micronutrient deficiencies in young infants. In addition, this is one of the few studies that has data on both infant and maternal micronutrient status for infants at three months. 

## 5. Conclusions

Overall, we found a significant proportion of infants were either deficient or marginally deficient in vitamin D and vitamin B12, particularly among infants at two weeks, and that maternal status for vitamin D and vitamin B12 were significant predictors of infant micronutrient deficiency for infants at three months. Further research is necessary to determine the burden of micronutrient deficiencies in infancy in other settings, and to examine the potential benefits of improving infants’ status through incorporating vitamin D and vitamin B12 supplements into the currently provided routine prenatal supplements or through direct infant supplementation. 

## Figures and Tables

**Table 1:** Definitions and cutoff values for maternal and infant micronutrient status.

Indicator	Definition	Population	Cutoff
Serum 25(OH)D	Vitamin D Deficiency [34]	All Ages	<50 nmol/L
	Insufficient Vitamin D [34]	All Ages	50–75 nmol/L
Serum Vitamin B12	Vitamin B12 Deficiency [18]	All Ages	<203 pg/mL
	Insufficient Vitamin B12 [18]	All Ages	203–271 pg/mL
Serum Folate	Folate Deficiency [18]	All Ages	<4 ng/ml
Serum Ferritin	Iron Deficiency	Adults [35]	<15 ng/mL
		Infants 3 months [35]	<12 ng/mL
		Infants 2 weeks [36]	<30 ng/mL

**Table 2:** Infant and maternal baseline characteristics for infants.

	Infants 2 Weeks (*n* = 446)	Infants 3 Months (*n* = 408)
**Infant Characteristics**		
Age (weeks) (mean (SD))	2.4 (0.3)	13.2 (0.5)
Male (*n* (%))	231 (51.8)	214 (52.5)
Preterm (<37 weeks) (*n* (%))	51 (13.1)	50 (16.9)
Birthweight (g) (mean (SD))	3084.3 (449.5)	3063.3 (475.6)
Small for gestational age (*n* (%))	68 (17.4)	56 (19.0)
Twin or triplet (*n* (%))	8 (1.8)	13 (3.2)
Cesarean section (*n* (%))	26 (5.8)	18 (4.4)
Breastfed within 1 h of birth (*n* (%))	395 (88.6)	374 (91.7)
Fed Colostrum (*n* (%))	435 (97.5)	401 (98.3)
Breastfeeding status at 3 months (*n* (%))		
Exclusive	^1^	175 (42.9)
Predominant/Partial	^1^	189 (46.3)
None	^1^	8 (2.0)
Unknown/missing	^1^	36 (8.8)
Randomized to Vitamin A group (*n* (%))	215 (48.2)	214 (52.4)
Residency ^2^		
Urban (*n* (%))	66 (14.8)	141 (34.6)
Rural (*n* (%))	380 (85.2)	267 (65.4)
**Maternal Characteristics**		
Maternal Age (mean (SD))	25.0 (5.8)	26.4 (6.3)
First Pregnancy (*n* (%))	98 (22.0)	84 (20.6)
Maternal Education (*n* (%))		
None	35 (7.9)	34 (8.3)
Primary	359 (80.5)	314 (77.0)
Secondary+	39 (8.7)	46 (11.2)
Missing	13 (2.9)	14 (3.43)
Provided Maternal Vitamin A Supplement (*n* (%))	142 (31.9)	111 (27.2)
Antenatal Care Visits ≥ 4 (*n* (%)) ^3^	103 (23.1)	103 (25.3)

^1^ Not available for infants at 2 weeks; ^2^ Urban = Dar es Salaam, Rural = Morogoro; ^3^ Recommended Antenatal Care visits based on WHO guidelines at the time of recruitment. Abbreviations: Standard Deviation: SD.

**Table 3:** Descriptive statistics for infant and mothers micronutrient status.

	Infants Two Weeks			Infants Three Months			Mothers Three Months Postpartum		
	*n*	Mean (SD)	Median (Q1, Q3) *	*n*	Mean (SD)	Median (Q1, Q3) *	*n*	Mean (SD)	Median (Q1, Q3) *
Vitamin D (nmol/L)	428	47.6 (14.8)	45.5 (37.1–55.6)	405	75.0 (21.5)	72.7 (60.6–86.0)	427	63.7 (14.8)	61.8 (53.3–71.6)
Vitamin B12 (pg/mL)	254	310.3 (154.0)	279.6 (197.3–378.9)	324	370.7 (190.8)	332.1 (248.3–434.8)	427	434.6 (202.3)	395.4 (286.8–523.5)
Folate (ng/mL)	253	15.5 (5.8)	14.3 (11.2–18.3)	324	20.9 (8.1)	19.4 (15.2–27.8)	427	7.3 (4.4)	6.1 (4.4–8.8)
Ferritin (ng/mL)	250	318.9 (192.9)	274.2 (192.2–393.8)	324	142.1 (165.2)	102.8 (59.9–170.2)	427	39.4 (44.2)	25.9 (14.6–47.7)

* Q1 = 25th percentile, Q3 = 75th percentile.

**Table 4:** Predictors for vitamin D deficiency (<50 nmol/L) for infants.

	Infants Two Weeks					Infants Three Months				
		Univariate		Multivariate			Univariate		Multivariate ^5^	
	*n* (%)	RR (95% CI)	*p* Value	RR (95% CI)	*p* Value	*n* (%)	RR (95% CI)	*p* Value	RR (95% CI)	*p* Value
Low Birthweight ^1^	28 (6.5)	1.03 (0.77, 1.37)	0.85	0.99 (0.75, 1.33)	1.00	31 (7.7)	3.02 (1.43, 6.34)	0.004	4.37 (1.95, 9.77)	0.0003
Preterm ^2^	50 (13.4)	0.95 (0.74, 1.21)	0.66	0.93 (0.73, 1.18)	0.57	50 (17.1)	1.48 (0.67, 3.26)	0.33	1.57 (0.72, 3.45)	0.26
Male	225 (52.6)	0.96 (0.83, 1.11)	0.56	0.97 (0.84, 1.11)	0.63	212 (52.4)	0.96 (0.51, 1.82)	0.91	0.97 (0.53, 1.77)	0.92
Urban Residence ^3^	66 (15.4)	1.05 (0.86, 1.27)	0.63	1.10 (0.90, 1.34)	0.37	141 (34.8)	1.98 (1.06, 3.72)	0.03	2.95 (1.46, 5.97)	0.003
ANC < 4 ^4^	232 (69.9)	1.00 (0.83, 1.2)	0.99	1.03 (0.85, 1.23)	0.79	218 (67.9)	1.08 (0.46, 2.54)	0.86	1.21 (0.52, 2.81)	0.65
Maternal Education										
None	34 (8.2)	Ref (1.00)		Ref (1.00)		34 (8.7)	Ref (1.00)		Ref (1.00)	
Primary (1–7)	342 (82.4)	1.57 (1.10, 2.24)	<0.0001 *	1.58 (1.10, 2.26)	<0.0001 *	312 (79.8)	4.46 (0.62, 32.00)	0.17 *	5.15 (1.04, 25.53)	0.03 *
Secondary+ (>7)	39 (9.4)	2.03 (1.39, 2.96)		2.05 (1.40, 3.00)		45 (11.5)	5.33 (0.65, 43.91)		8.45 (1.45, 49.18)	
Maternal Age (years)										
<20	78 (18.2)	Ref (1.00)		Ref (1.00)		65 (16.1)	Ref (1.00)		Ref (1.00)	
20–25	171 (40.0)	0.86 (0.72, 1.04)	0.52	0.84 (0.70, 1.01)	0.70	129 (31.9)	0.71 (0.23, 2.14)	0.37 *	0.55 (0.16, 1.85)	0.16 *
26–29	81 (18.9)	0.79 (0.62, 1.00)		0.80 (0.63, 1.02)		83 (20.5)	1.88 (0.70, 5.07)		1.95 (0.72, 5.32)	
30+	98 (22.9)	0.93 (0.76, 1.14)		0.94 (0.77, 1.14)		128 (31.6)	1.12 (0.41, 3.08)		1.21 (0.45, 3.26)	
Maternal Vitamin D Status										
Maternal Vitamin D >50nmol/L	n/a	n/a		n/a		307 (75.8)	Ref (1.00)		Ref (1.00)	
Maternal Vitamin D <50nmol/L	n/a	n/a		n/a		54 (15.0)	3.29 (1.66,6.52)	0.0006	2.83 (1.45, 5.52)	0.002

^1^ <2500 g; ^2^ <37 weeks; ^3^ Reference group: Rural residence. Urban defined as those residing in Dar es Salaam. Rural defined as those residing in Morogoro; ^4^ Reference Group: Those who received four or more ANC visits; ^5^ Includes all covariates run in the univariate model. Preterm and low birthweight were not included in the same model. For all covariates besides preterm, reported RR and *p* values are from model that included low birthweight. For preterm covariates, RR and p values were reported from multivariate models without low birthweight; * *p* for trend. Abbreviations: Reference: Ref.

**Table 5:** Predictors for vitamin B12 deficiency for infants.

	Infants Two Weeks					Infants Three Months				
		Univariate		Multivariate ^6^			Univariate		Multivariate ^6^	
	*n* (%)	RR (95% CI)	*p* Value	RR (95% CI)	*p* Value	*n* (%)	RR (95% CI)	*p* Value	RR (95% CI)	*p* Value
Low Birthweight ^1^	18 (7.1)	0.83 (0.34, 2.03)	0.69	0.93 (0.42, 2.07)	0.86	26 (8.0)	1.51 (0.65, 3.50)	0.34	1.06 (0.43, 2.56)	0.89
Preterm ^2^	30 (14.3)	0.75 (0.35, 1.59)	0.45	0.85 (0.39, 1.83)	0.67	31 (13.6)	0.71 (0.23, 2.19)	0.55	0.65 (0.24, 1.79)	0.41
Male	140 (55.1)	0.89 (0.59, 1.34)	0.58	0.87 (0.58, 1.30)	0.49	176 (54.3)	1.29 (0.73, 2.28)	0.39	1.11 (0.65, 1.91)	0.70
Urban Residence ^3^	62 (24.4)	0.68 (0.39, 1.18)	0.17	0.64 (0.36, 1.14)	0.13	140 (43.2)	0.63 (0.35, 1.15)	0.14	1.01 (0.57, 1.80)	0.98
ANC < 4 ^4^	142 (69.6)	1.04 (0.63, 1.71)	0.89	0.99 (0.60, 1.63)	0.97	171 (64.5)	1.47 (0.71, 3.02)	0.30	1.68 (0.84, 3.37)	0.14
Maternal Education										
None	20 (8.2)	Ref (1.00)		Ref (1.00)		27 (8.6)	Ref (1.00)		Ref (1.00)	
Primary (1–7)	197 (80.4)	1.25 (0.59, 2.65)	0.20 *	1.32 (0.64, 2.73)	0.12 *	248 (79.0)	0.82 (0.37, 1.82)	0.55 *	0.76 (0.39, 1.49)	0.86 *
Secondary + (>7)	28 (11.4)	1.73 (0.72, 4.12)		1.84 (0.79, 4.25)		39 (12.4)	0.63 (0.19, 2.06)		0.94 (0.30, 2.88)	
Maternal Age (years)										
<20	38 (15.0)	Ref (1.00)		Ref (1.00)		53 (16.4)	Ref (1.00)		Ref (1.00)	
20–25	112 (44.1)	2.21 (0.82, 5.91)	0.001 *	2.29 (0.84, 6.22)	0.0006	104 (32.1)	0.31 (0.14, 0.71)	0.23 *	0.45 (0.20, 1.02)	0.18 *
26–29	47 (18.5)	3.23 (1.18, 8.87)		3.44 (1.22, 9.71)		67 (20.7)	0.67 (0.33, 1.37)		1.01 (0.51, 2.00)	
30+	57 (22.4)	3.50 (1.30, 9.39)		3.66 (1.35, 9.92)		100 (30.9)	0.45 (0.22, 0.93)		0.49 (0.24, 1.01)	
Maternal Vitamin B12 Status ^5^										
Maternal Vitamin B12 Sufficiency	n/a	n/a		n/a		227 (70.1)	Ref (1.00)		Ref (1.00)	
Maternal Vitamin B12 Insufficiency	n/a	n/a		n/a		48 (16.6)	3.72 (1.80, 7.68)	0.0004	4.06 (1.88, 8.70)	0.0003
Maternal Vitamin B12 Deficiency	n/a	n/a		n/a		14 (4.8)	9.27 (4.69, 18.30)	<0.0001	9.63 (4.83, 19.17)	<0.0001

^1^ <2500 g; ^2^ <37 weeks; ^3^ Reference group: Rural residence. Urban defined as those residing in Dar Es Salaam. Rural defined as those residing in Morogoro; ^4^ Reference Group: Those who received four or more ANC visits; ^5^ Vitamin B12 Cutoffs are displayed in Table 1; ^6^ Includes all covariates run in the univariate model. Preterm and low birthweight were not included in the same model. For all covariates besides preterm, reported RR and p values are from model that included low birthweight. For preterm covariates, RR and *p* values were reported from multivariate models without low birthweight; * *p* for trend.
